# Prediction of stroke recurrence for patients with middle cerebral artery atherosclerotic disease by CT perfusion

**DOI:** 10.3389/fneur.2025.1739005

**Published:** 2026-01-07

**Authors:** Xi Zhang, Zhi Li, He Zhang, Zhibin Chen, Guangxin Duan, Shi Huang, Hongchao Shi, Yunfeng Zhang, Junhao Du, Guodong Xiao, Yun Luo

**Affiliations:** 1 Department of Neurology, Nanjing University Medical School Affiliated Nanjing Drum Tower Hospital, Nanjing, China; 2 Department of Stroke Center, Affiliated Hospital of Nantong University, Nantong, China; 3 Department of Neurology, Nanjing First Hospital, Nanjing Medical University, Nanjing, China; 4 Department of Neurology, The Second Affiliated Hospital of Soochow University, Suzhou, China

**Keywords:** CTP, ICAD, middle cerebral artery, recurrence, stroke

## Abstract

**Background:**

This paper aimed to test the feasibility of computed tomography perfusion (CTP) in predicting stroke recurrence in patients with symptomatic middle cerebral artery atherosclerotic disease.

**Methods:**

This is a retrospective study. 322 patients from 4 advanced stroke centers diagnosed as symptomatic middle cerebral artery atherosclerotic disease, including severe stenosis and occlusion were recruited. All patients underwent head CTP, digital subtraction angiography (DSA), MR 14-21 days after onset. Stroke recurrence of patients within 3 months was recorded. Patients were divided into recurrence group and control group according to whether stroke recurrence 3 months after onset. The association of imaging characteristics, other risk factors and patients stroke recurrence was assessed.

**Results:**

104 patients presented stroke recurrence within 3 months after onset. Patients in recurrence group showed more severe vascular stenosis (*p* < 0.001) and presented with larger hypoperfusion area than control group, as shown by larger Tmax>4 s, Tmax>6 s, Tmax>8 s, Tmax>10s volume (*p* < 0.001). The multiple logistic regression showed that the volume of Tmax>4 s (OR = 1.061 per 10 mL increase, 95% CI: 1.005–1.122, *p* = 0.033) and Tmax>6 s (OR = 1.265 per 10 mL increase, 95% CI: 1.093–1.483, *p* = 0.002) at 14–21 days after stroke were strongly associated with stroke recurrence, Tmax>4 s (AUC = 0.76, 95% CI: 0.70 to 0.81, *p* < 0.001) and Tmax>6 s (AUC = 0.74, 95% CI: 0.68 to 0.81, p < 0.001) volume had a high discriminative ability for a poor outcome. Tmax>4 s volume larger than 133.60 mL being an optimal cutoff (sensitivity = 80.7% and specificity = 58.6%, Youden index = 39.3%) and Tmax>6 s volume larger than 17.95 mL being an optimal cutoff (sensitivity = 83.5% and specificity = 58.7%, Youden index = 42.2%).

**Conclusion:**

Symptomatic middle cerebral artery atherosclerotic stenosis or occlusion patients with severe hypoperfusion might suffer a higher risk of stroke recurrence. Volume of Tmax>4 s and Tmax>6 s in CTP could be used to predict the prognosis of these patients.

## Introduction

Ischemic stroke is the leading cause of death and disability of Chinese people. The latest epidemiological data show that there are as many as 10.3 million stroke patients per year, with ischemic stroke accounting for approximately 80–85% (1, 2). Intracranial atherosclerotic disease (ICAD) is one of the important causes of ischemic stroke occurrence and recurrence worldwide (3, 4). In North America, ICAD accounts for 8 to 10% of stroke etiologies, while in Asia, it accounts for 30 to 50% (5, 6). In China, the incidence of ICAD among stroke/TIA patients is as high as 46.6% (7, 8). ICAD increases the risk of stroke occurrence and recurrence, it is also one of the important risk factors for poor prognosis.

The middle cerebral artery is the largest intracranial branch of the internal carotid artery. Once occluded, it can lead to severe cerebral infarction, resulting in disability or even life-threatening consequences for the patients. Among Chinese population, the middle cerebral artery (MCA) is the most affected intracranial large artery by ICAD (9), with an incidence rate of 33 to 37%. Studies have shown that patients with symptomatic MCA atherosclerotic stenosis have an annual stroke risk of up to 14.3%, and for those with hemodynamic impairment, the annual stroke risk is doubled (10). However, the pathophysiological mechanisms underlying the high recurrence of stroke in these patients remain unclear. There are two possible mechanisms that contribute to ischemic events: 1) hemodynamic impairment; 2) impaired thrombus clearance (11, 12).

After MCA atherosclerotic disease, the blood flow decreases, leading to insufficient perfusion of the brain tissue in the supplied area. Long-term hypoperfusion prompts various vascular compensatory mechanisms to maintain blood and oxygen supply to the brain tissue (12). However, this compensation is often unstable, and sudden blood pressure drops or abrupt temperature changes can instantly disrupt the fragile compensation, leading to severe hypoperfusion and ultimately inducing cerebral infarction. Patients with this pathogenesis often present with watershed cerebral infarctions. At the same time, severe stenosis can reduce blood flow velocity, and the damaged endothelium at the stenosis site can promote thrombus formation. When patients’ thrombus clearance capacity is impaired, severe embolic events occur (13). Under this mechanism, patients mostly present with perforator infarctions, cortical embolisms, or large-area cerebral infarctions. And these mechanisms often coexist.

For symptomatic ICAD, pharmacological therapy still dominates. However, even with optimal medical therapy, the annual stroke risk for these patients remains as high as 10.4% (14), ultimately leading to severe disability or even death. Currently, the Chinese expert consensus on intracranial artery stenosis indicates that patients who experience two stroke episodes while on medical therapy may be considered for EVT. However, many patients suffer severe cerebral infarction during the second stroke, and even with EVT, their prognosis remains poor. And although EVT could solve the risk caused by hemodynamic impairment (15), how to select patients who might benefit from it, which is the gap our study aims to fill. In the DEFUSE3 study (16), volume of Tmax > 6 s CTP was used to define hypoperfusion area of acute ischemic stroke patients (17). A few single-center retrospective studies have found that CT perfusion imaging can be used to predict stroke recurrence in patients with anterior circulation ICAD. It appears that the volume of Tmax >4 s is closely related to stroke recurrence, but these studies were limited by small sample sizes and single-center designs, precluding definitive conclusions (18–20).

In this paper, we reviewed the data from four medical centers, aimed to test the feasibility of computed tomography perfusion (CTP) in predicting stroke recurrence in patients with symptomatic middle cerebral artery atherosclerotic disease.

## Method

The data that support the findings of this study are available from the corresponding author upon reasonable request. The institutional review board approved this study and IRB (Ethics Committee of Nanjing Drum Tower Hospital) number: 2021–399-02. Informed consent was obtained from all the patients.

### Patients

**Figure 1:**
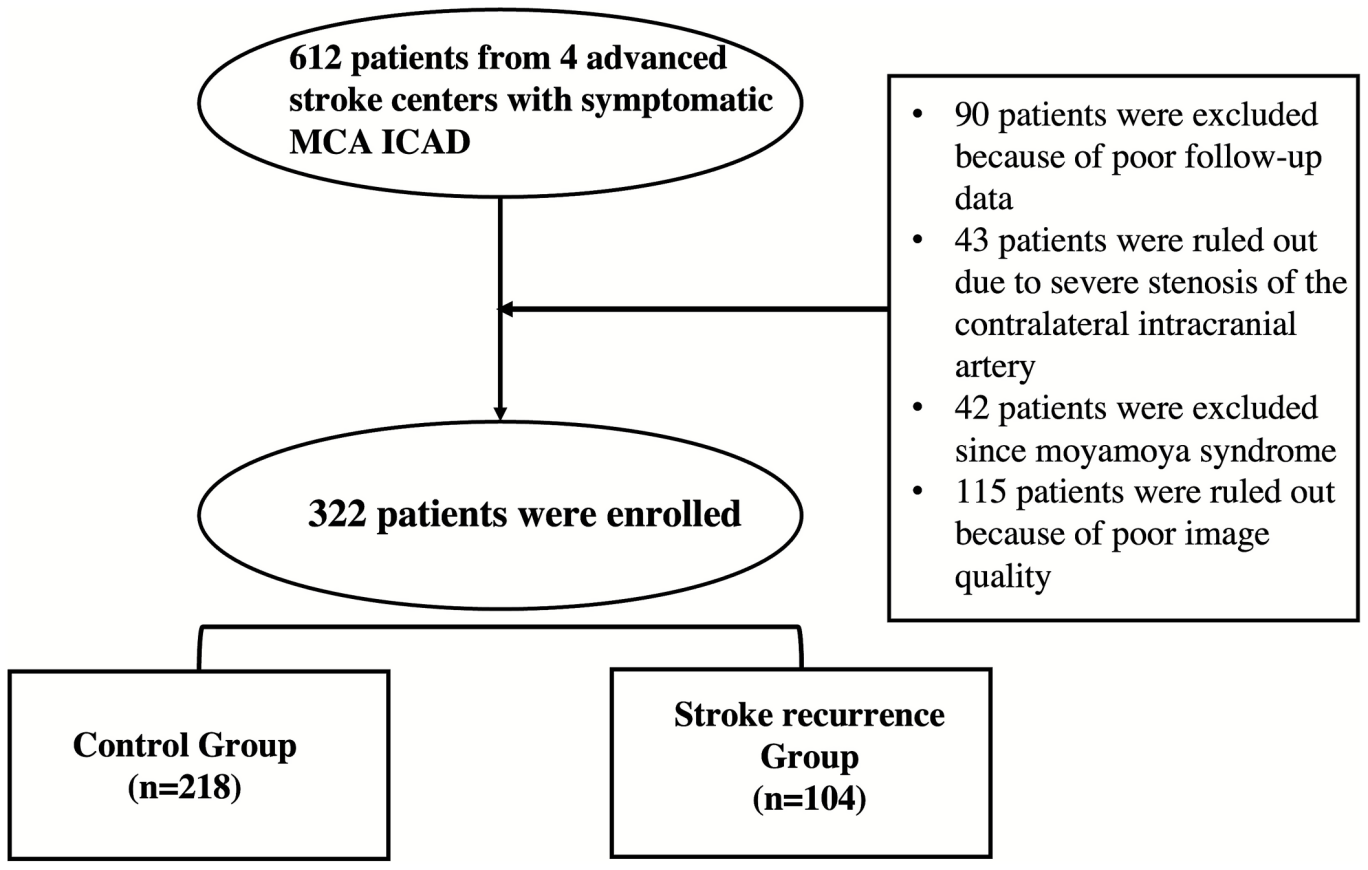
Flow chart of the study.

From Jan 1, 2021, to Sep 30, 2024, a total of 612 patients in 4 advanced stroke centers with symptomatic MCA atherosclerotic disease were reviewed. 90 patients were excluded because of poor follow-up data, 43 patients were ruled out due to severe stenosis of the contralateral intracranial artery, 42 patients were excluded since moyamoya syndrome, 115 patients were ruled out because of poor image quality (shown in Figure 1). Finally, 322 patients were enrolled. All patients underwent head CTP, digital subtraction angiography (DSA), high-resolution vessel wall MR 14–21 days after onset. Stroke recurrence of patients within 3 month was recorded. Patients were divided into recurrence group and control group according to whether stroke recurrence 3 month after onset.

The selection criteria of patients were as follows: 1) The stenosis degree of MCA was confirmed by DSA, stenosis severity must exceed 70%; 2) MCA atherosclerotic disease confirmed by high-resolution vessel wall MR; 3) presenting with related stroke; 4) ipsilateral intracranial and extracranial artery stenosis <50%; 5) contralateral intracranial and extracranial artery stenosis <70%. The exclusion criteria were as follows: 1) clinical, laboratory, or imaging findings not suspicious for atherosclerotic lesions, such as vasculitis, Moyamoya syndrome, arterial dissection; 2) a coexisting cardioembolic source (eg, mitral stenosis, prosthetic valve, myocardial infarction within 6 weeks, intracardiac clot, ventricular aneurysm, and bacterial endocarditis); 3) a concomitant intracranial aneurysm or any bleeding disorder; 4) large infarct core, defined as an Alberta Stroke Program Early CT Score (ASPECTS) of < 6 points.

### Clinical assessment

We collected baseline data from patients, including age, gender, risk factors for stroke, and the degree of vascular stenosis. Stroke recurrence was defined as the onset of new clinical symptoms or worsening of pre-existing symptoms, which were attributable to the culprit vessel and confirmed by DWI sequence. The measurement of the degree of vascular stenosis was performed by two independent, qualified clinicians. Intraclass Correlation Coefficient (2, 1) = 0.91.

### Imaging acquisition

All patients underwent DSA and MRI after onset to confirm whether cerebral infarction was caused by middle cerebral artery atherosclerosis. CTP was performed within 14–21 days after onset to assess intracranial perfusion status. High-resolution vessel wall MR was taken to differentiate whether the stenosis is caused by atherosclerosis. Subsequently, a 3-month follow-up was conducted. Patients who experienced stroke recurrence immediately underwent cranial MRI to evaluate lesion characteristics.

### Secondary prevention of ischemic stroke

All patients received oral dual antiplatelet therapy (conventionally aspirin combined with clopidogrel, for patients could not tolerate clopidogrel, Cilostazol was taken into consideration), lipid-lowering and plaque-stabilizing therapy with statins, as well as blood pressure and glucose control. They are also advised to improve their lifestyle habits, such as quitting smoking and alcohol, and maintaining regular exercise.

### Statistical analysis

Median and interquartile range were used to describe continuous variables. The count (n) and percentage (%) were used to describe categorical variables. Baseline and procedural characteristics and treatment outcomes of recurrence group and control group were compared. The categorical and binary variables were analyzed by χ^2^ test, and continuous variables were analyzed by Mann–Whitney U test. ROC curves were used to evaluate the prediction performance of Tmax> 4 s volume and Tmax> 6 s volume.

In addition, a multinomial logistic regression analysis was performed to assess the correlation between hypoperfusion and stroke recurrence, the covariates were degree of stenosis, volume of Tmax>4 s, volume of Tmax>6 s, volume of Tmax>8 s and volume of Tmax>10s, the assumptions of logistic regression was checked by Hosmer-Lemeshow and Tjur’s R squared analysis. VIF was taken to exam the Multicollinearity of covariates, VIF < 5 was taken into consideration. Youden index was used to find the optimal cutoff point, selecting the largest Youden index as the best cutoff. All statistical analyses were completed by SPSS software (version 25.0; IBM SPSS, Chicago, IL), and *p* value <0.05 was considered significant.

## Results

This study is retrospective research. From Jan 1, 2021 to Sep 30, 2024 a total of 612 patients from 4 advanced stroke centers with symptomatic MCA atherosclerotic disease were reviewed. Finally, 322 patients were enrolled. These patients were divided into 2 groups according to whether they experienced stroke recurrence within 3 months after their initial onset. Of which 104 patients in the recurrence group and 218 patients in the control group.

As shown in Table 1, the overall mean age was 60.0 years (IQR 54–69), and 117 (36.3%) participants were female. Medical history including hypertension, diabetes, dyslipidemia, previous ischemic stroke or TIA, myocardial infarction, baseline NIHSS score and baseline mRS score showed no significant difference. Patients in recurrence group showed more severe MCA stenosis (*p* = 0.03). Also patients with stroke recurrence presented more severe intracranial hypoperfusion, which reflected in larger volume of Tmax>4 s (153.00 ± 8.78 vs. 19.95 ± 4.42, *p* = 0.001) and Tmax>6 s (47.19 ± 5.15 vs. 10.26 ± 1.41, p = 0.001) in CTP. We analyzed the patients’ 3-month mRS and NIHSS scores and found no statistically significant differences between the control group and the recurrence group.

**Table 1:** Study population characteristics.

Variable	Total	Recurrence group	Control group	*P* value
Patients, *n*	322	104	218	
Female sex, *n* (%)	117 (36.3%)	34 (32.7%)	83 (38.1%)	0.35
Median age (IQR), y	60.0 (54,69)	63.5 (55, 70)	59 (53, 69)	0.16
Medical history				
Hypertension, *n* (%)	242 (75.2%)	77 (74.0%)	165 (75.7%)	0.75
Stable blood pressure control	298 (92.5%)	97 (93.2%)	201 (92.2%)	0.82
Diabetes, *n* (%)	30 (9.3%)	9 (8.7%)	21 (9.6%)	0.84
Stable blood glucose control	294 (91.3%)	95 (91.3%)	199 (91.3%)	0.99
Dyslipidemia, *n* (%)	159 (49.4%)	54 (51.9%)	105 (48.2%)	0.53
Previous ischemic stroke or TIA, *n* (%)	68 (21.1%)	22 (21.2%)	46 (21.1%)	0.99
Myocardial infarction, *n* (%)	11 (3.4%)	3 (2.9%)	8 (3.7%)	0.99
Smoking, *n* (%)	161 (50.0%)	48 (46.2%)	113 (51.8%)	0.34
Quit smoking after stroke	209 (64.9%)	64 (61.5%)	145 (66.5%)	0.39
Baseline NIHSS score (IQR)	3 (2, 4)	3 (2, 4)	3 (2, 4)	0.77
Baseline mRS score (IQR)	1 (1, 1)	1 (1, 1)	1 (1, 1)	0.93
Oral antiplatelet medication	322 (100%)	104 (100%)	218 (100%)	0.99
90 days NIHSS score (IQR)	2 (1, 3)	2 (1, 3)	2 (1, 3)	0.54
90 days mRS score (IQR)	1 (1, 1)	1 (1, 1)	1 (1, 1)	0.81
Degree of stenosis				0.03
70–79%, *n* (%)	57 (17.7%)	12 (11.5%)	45 (20.6%)	
80–99%, *n* (%)	156 (48.4%)	48 (46.2%)	108 (49.5%)	
100%, *n* (%)	109 (33.9%)	44 (42.3%)	65 (29.8%)	
Volume of Tmax>4 s (Mean±SE), ml	103.60 ± 4.54	153.00 ± 8.78	19.95 ± 4.42	0.001
Volume of Tmax>6 s (Mean±SE), ml	22.19 ± 2.14	47.19 ± 5.15	10.26 ± 1.41	0.001
Volume of Tmax>8 s (Mean±SE), ml	7.14 ± 1.17	17.70 ± 3.17	2.11 ± 0.59	0.001
Volume of Tmax>10s (Mean±SE), ml	2.57 ± 0.63	6.42 ± 1.79	0.72 ± 0.28	0.001

**Figure 2:**
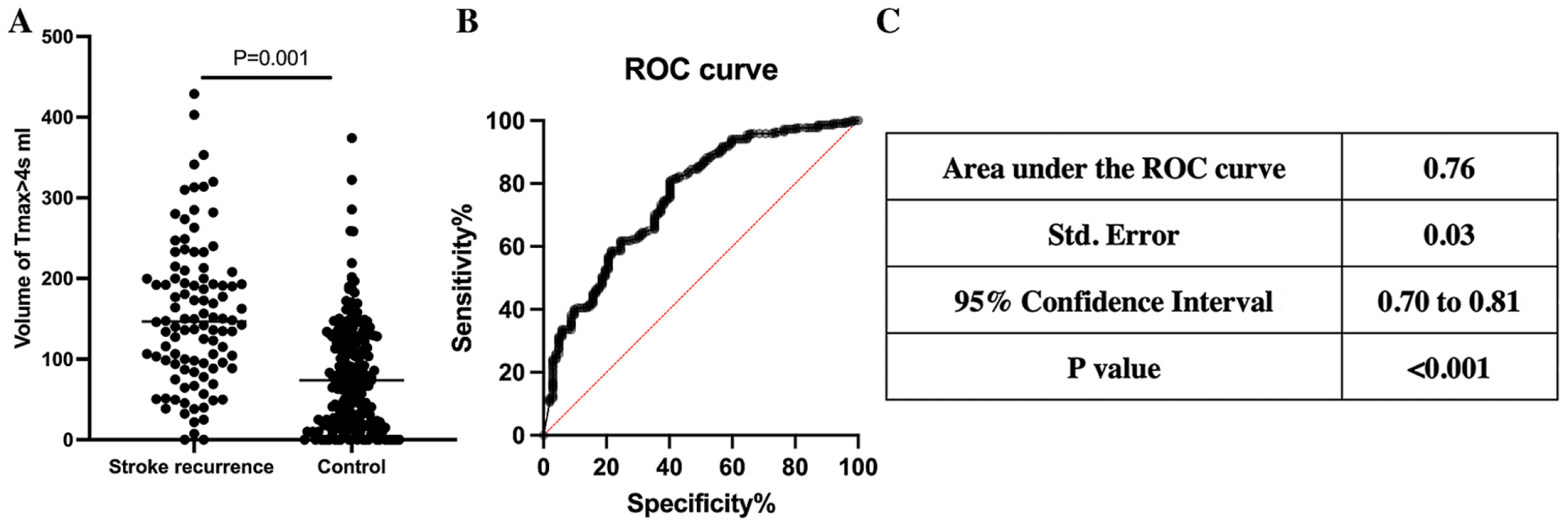
Tmax > 4 s volume in CTP and stroke recurrence. **(A)** Comparison of Tmax >4 s volumes between the control group and the recurrence group, the recurrence group had a larger Tmax >4 s volume (*p* = 0.001). **(B)** ROC curve of Tmax > 4 s volume and stroke recurrence. **(C)** Details of ROC curve, AUC = 0.76, *p* < 0.001.

**Figure 3:**
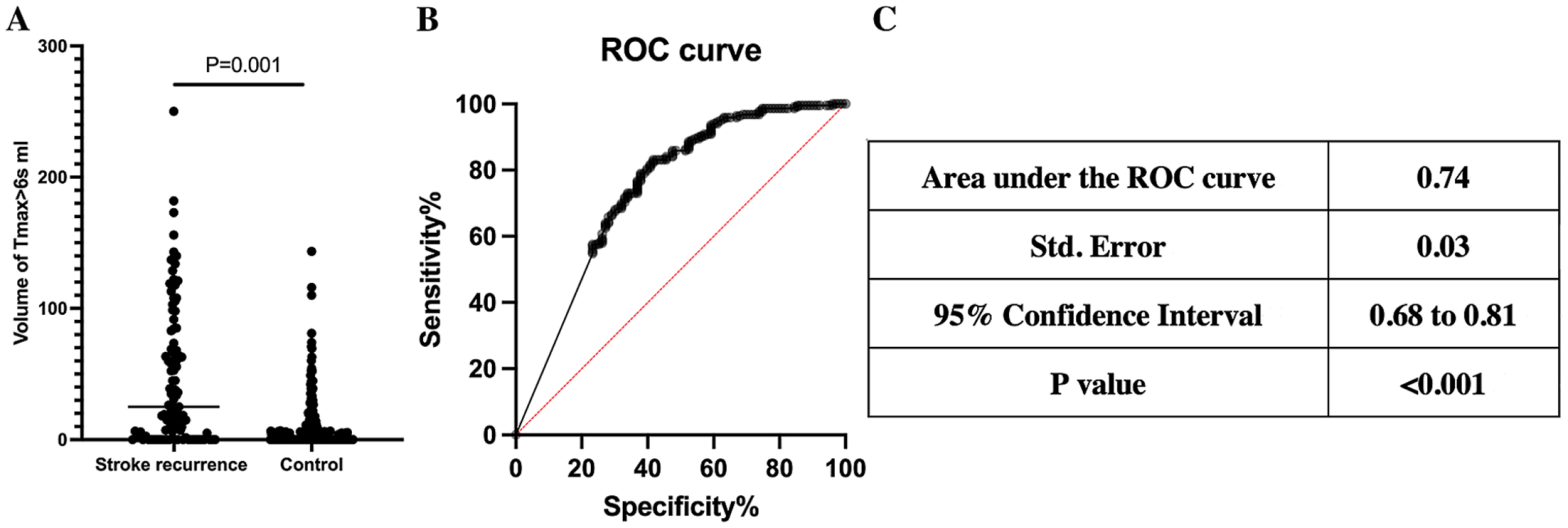
Tmax>6 s volume in CTP and stroke recurrence. **(A)** Comparison of Tmax >6 s volumes between the control group and the recurrence group, the stroke recurrence group had a larger Tmax >6 s volume (*p* = 0.001). **(B)** ROC curve of Tmax>6 s volume and stroke recurrence. **(C)** Details of ROC curve, AUC = 0.74, *p* < 0.001.

Multiple factor logistic regression analysis showed that volume of Tmax>4 s (OR = 1.061 per 10 mL increase, 95% CI: 1.005–1.122, *p* = 0.033) and Tmax>6 s (OR = 1.265 per 10 mL increase, 95% CI: 1.093–1.483, *p* = 0.002) volume of MAC ICAD patients were highly correlated with stroke recurrence, which were shown in Table 2. ROC analysis showed that Tmax>4 s volume (AUC = 0.76, 95% CI: 0.70 to 0.81, *p* < 0.001) and Tmax>6 s volume (AUC = 0.74, 95% CI: 0.68 to 0.81, p < 0.001) had a high discriminative ability for a poor outcome. Tmax>4 s volume larger than 133.60 mL being an optimal cutoff (sensitivity = 80.7% and specificity = 58.6%, Youden index = 39.3%) and Tmax>6 s volume larger than 17.95 mL being an optimal cutoff (sensitivity = 83.5% and specificity = 58.7%, Youden index = 42.2%) (Figures 2, 3).

**Table 2:** Multiple factor logistic regression analysis of stroke recurrence.

Variable	OR	95% CI	*P* value
Degree of stenosis	0.985	0.956–1.014	0.312
Volume of Tmax>4 s	1.061	1.005–1.122	0.033
Volume of Tmax>6 s	1.265	1.093–1.483	0.002
Volume of Tmax>10s	0.884	0.598–1.421	0.562

**Figure 4:**
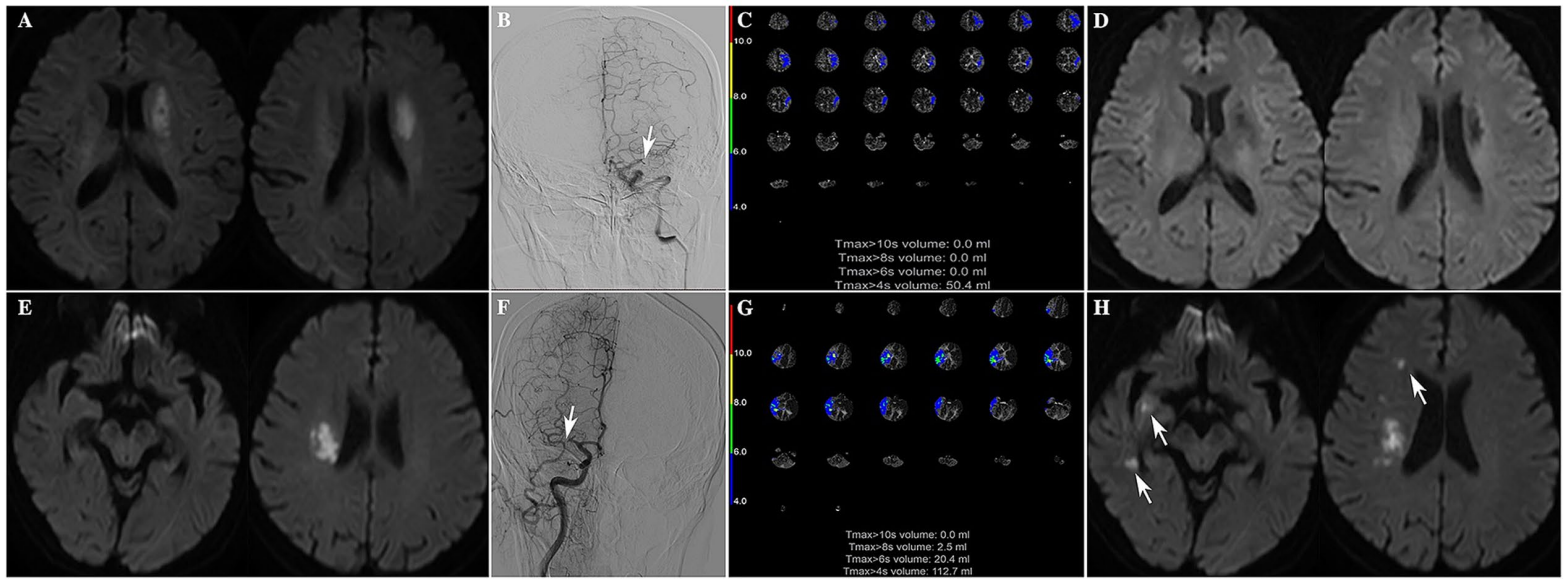
One representative case from the control group and one from the recurrence group. **(A)** MR DWI sequence shows a subacute cerebral infarction lesion in the left basal ganglia region. **(B)** Occlusion of the M1 segment of the left MCA in DSA (indicated by white arrow). **(C)** CTP indicates a Tmax > 4 s volume of 50.4 mL. **(D)** A follow-up magnetic resonance DWI sequence performed 3 months after onset shows an old cerebral infarction in the left basal ganglia, with no new infarction lesions observed. **(E)** MR DWI sequence shows an acute cerebral infarction lesion in the right basal ganglia region. **(F)** Occlusion of the M1 segment of the right MCA in DSA (indicated by white arrow). **(G)** CTP indicates a Tmax > 4 s volume of 112.7 mL and a Tmax > 6 s volume of 20.4 mL. **(H)** A follow-up magnetic resonance DWI sequence performed 1 month after onset shows new cerebral infarction lesions in the right basal ganglia and right temporal lobe (indicated by white arrows).

Also, we presented one case from recurrence group and one case from the control group in Figure 4 to visually illustrate the association between CTP-derived Tmax abnormalities and clinical outcomes.

## Discussion

This study found that intracranial perfusion status was significantly associated with stroke recurrence in patients with middle cerebral artery atherosclerotic disease. We evaluated intracranial hypoperfusion using Tmax >4 s volume and Tmax >6 s volume. The results demonstrated that larger ischemic volumes correlated with higher risks of stroke recurrence, which is consistent with the pathogenesis of intracranial atherosclerotic disease (ICAD) patients. This finding is consistent with the results reported in the paper by Yu et al. (21).

Our further analysis revealed that ICAD patients with either Tmax>4 s volumes exceeding 133.60 mL or Tmax>6 s volumes surpassing 17.95 mL demonstrated significantly elevated stroke risks. However, these findings require validation in larger patient cohorts. We analyzed the patients’ 3-month mRS and NIHSS scores and found no statistically significant differences between the control group and the recurrence group. We offer the following possible explanations for these results: 1. The patients were still in the recovery period; extending the follow-up to 1 year might yield different findings. 2. Based on the baseline mRS and NIHSS scores, most patients had mild stroke severity, and even in cases of recurrence, many did not experience significant disability, which differs from patients with acute intracranial artery occlusion. In subsequent studies, we will extend the follow-up duration and reevaluate these outcomes.

Compared to previous single-center retrospective studies, our research presents the following distinctions: 1. We incorporated data from four advanced stroke centers, making this a multicenter retrospective analysis. 2. We screened 612 patients and included 322, resulting in a sample size substantially larger than those of prior studies. 3. We focused specifically on patients with atherosclerotic lesions in the middle cerebral artery, thereby avoiding the influence of the Circle of Willis on perfusion measurements. By excluding cases with penetrating artery disease, the patient cohort is more homogeneous, which enhances the reliability of the findings. 4. Perfusion results for all patients were analyzed using the F-stroke software, reducing bias that may arise from differences in analytical methods.

Currently, endovascular treatment for symptomatic ICAD remains in its early stages, with no positive results from large-scale randomized controlled trials (RCT) available either domestically or internationally to support its efficacy. The results of SAMMPRIS and VISSIT study showed that stenting had higher complication rates and worse patient outcomes compared to medical therapy (22–25). However, there have been numerous academic questions and criticisms surrounding these two studies. The CASSISS study proposed two important improvements: (1) selecting more experienced neurointerventionalists to reduce perioperative complications; and (2) extending the follow-up period to 3 years to observe the long-term prognosis of endovascular treatment for intracranial artery stenosis (26, 27). But even with these improvements, the CASSISS study ultimately resulted in negative findings. The study showed no significant difference in the risk of stroke or death within 30 days and the risk of stroke in the responsible vascular territory within 1 year between patients who received stenting in addition to medical therapy and those who received medical therapy alone. Furthermore, during the subsequent 7-year follow-up period in the CASSISS study, stenting combined with medical therapy still failed to demonstrate any benefit (28).

Although existing studies failed to provide conclusive evidence that endovascular treatment benefits ICAD patients, a subset of these patients do demonstrate significant clinical improvement after the procedure. Therefore, developing reliable criteria for selecting appropriate ICAD candidates for endovascular therapy has become an urgent clinical priority (29).

Our study evaluated the degree of hypoperfusion in ICAD patients using CTP, investigated the correlation between hypoperfusion and stroke recurrence, and ultimately quantified these findings to establish a theoretical framework for selecting candidates suitable for endovascular therapy.

However, our study still has several limitations: 1. To avoid interference from the Circle of Willis, we focused solely on the middle cerebral artery in this study; 2. As a retrospective study, patients with incomplete data were excluded; 3. Despite being a multicenter study, the sample size remains relatively small; 4. The observation period was short, focusing only on stroke recurrence within 3 months; 5. Hypoperfusion was assessed solely by CTP, and few stroke recurrence-related risk factors were included. In the next phase, we will conduct a multicenter, prospective RCT to further investigate the relationship between intracranial hypoperfusion and stroke recurrence. These findings will undoubtedly provide a solid theoretical foundation for future endovascular treatment of ICAD.

## Conclusion

Symptomatic middle cerebral artery atherosclerotic stenosis or occlusion patients with severe hypoperfusion might suffer a higher risk of stroke recurrence. Volume of Tmax>4 s and Tmax>6 s in CTP could be used to predict the prognosis of these patients.

## Data Availability

The original contributions presented in the study are included in the article/supplementary material, further inquiries can be directed to the corresponding author/s.
